# Silicon Solar Cells on Glass with Power Conversion Efficiency above 13% at Thickness below 15 Micrometer

**DOI:** 10.1038/s41598-017-00988-x

**Published:** 2017-04-13

**Authors:** Paul Sonntag, Natalie Preissler, Matevž Bokalič, Martina Trahms, Jan Haschke, Rutger Schlatmann, Marko Topič, Bernd Rech, Daniel Amkreutz

**Affiliations:** 1 Institute for Silicon Photovoltaics, Helmholtz-Zentrum Berlin für Materialien und Energie GmbH, 12489 Berlin, Germany; 2 PVcomB, Helmholtz-Zentrum Berlin für Materialien und Energie GmbH, 12486 Berlin, Germany; 3 Faculty of Electrical Engineering, University of Ljubljana, 1000 Ljubljana, Slovenia; 4 Photovoltaics and Thin-Film Electronics Laboratory, Institute of Microengineering (IMT), Ecole Polytechnique Fédérale de Lausanne (EPFL), Lausanne, Switzerland

## Abstract

Liquid phase crystallized silicon on glass with a thickness of (10*–*40) μm has the potential to reduce material costs and the environmental impact of crystalline silicon solar cells. Recently, wafer quality open circuit voltages of over 650 mV and remarkable photocurrent densities of over 30 mA/cm^2^ have been demonstrated on this material, however, a low fill factor was limiting the performance. In this work we present our latest cell progress on 13 μm thin poly-crystalline silicon fabricated by the liquid phase crystallization directly on glass. The contact system uses passivated back-side silicon hetero-junctions, back-side KOH texture for light-trapping and interdigitated ITO/Ag contacts. The fill factors are up to 74% and efficiencies are 13.2% under AM1.5 g for two different doping densities of 1 · 10^17^/cm^3^ and 2 · 10^16^/cm^3^. The former is limited by bulk and interface recombination, leading to a reduced saturation current density, the latter by series resistance causing a lower fill factor. Both are additionally limited by electrical shading and losses at grain boundaries and dislocations. A small 1 × 0.1 cm^2^ test structure circumvents limitations of the contact design reaching an efficiency of 15.9% clearly showing the potential of the technology.

## Introduction

The past decade has witnessed severe price drops for (PV) modules causing a remarkable growth of the global installed capacity. Until the end of 2015, 242 GWp^1^ of PV capacity has been installed with a yearly growth rate of 30–50 GWp within the last few years. Assuming a specific silicon consumption of 6 g/Wp, 852.000 metric tons of silicon have been deployed since 2012. The energy demand for the fabrication of the raw silicon amounts to 362 TWh^2^ (specific energy consumption of 170 kWh/kg and 60% kerf-loss) which amounts to the total end energy demand of e.g. Germany's industry within one and a half years^3^. With module lifetimes exceeding 20 years, the energy demand during production is already on a low level, but still has to be improved. Liquid phase crystallized silicon on glass has the potential to further reduce the specific silicon consumption and therefore energy demand and costs of PV significantly while maintaining a wafer-like electronic quality^4^. In order to be competitive with the dominant multi-crystalline wafer technology, module efficiencies above 14% must be demonstrated for this technology^5^. One obstacle was the demonstration of a wafer equivalent (*V*_*OC*_) as an indicator for the electronic quality of the silicon absorber. As shown recently, using (LPC) technology *V*_*OC*_ above 650 mV can be realized using n-type material^6, 7^. In combination with established light trapping approaches (eg. potassium hydroxide (KOH) based texturing) and adapted contact systems, wafer-like efficiencies are feasible. Comparing the demonstrated efficiencies in this paper with other crystalline silicon thin-film approaches underlines the advantages of LPC technology. Jeong *et al*.^8^ realized a remarkable efficiency of 13.7% for a cell based on a nano-textured 10 μm mono-crystalline silicon absorber. However, for this demonstration, silicon-on-insulator technology was used, which is irrelevant for PV due to the high price of the wafers. In contrast to that, the absorbers in this work are formed directly on glass and only standard texturing was applied to increase the light-trapping inside the silicon. With an epitaxial foil concept Govaerts *et al*.^9^ reached 12.2% on a 40 μm thick layer of Si bonded to glass. However, the bonding process used in this work is still lithography based which is irrelevant for PV industry, but an anodization approach has been proposed. In contrast to the LPC-technology, this epitaxial foil bonding also uses mono-crystalline Si and the efficiency was achieved on an absorber three times as thick as in this work. Until now, the best reported efficiency on an LPC-Si absorber of 12.1% was limited by a fill factor below 69%^10^. In this paper we present improvements in device FF and our latest progress in cell development and efficiency improvement. The cells use an interdigitated back-contact (IBC) system on 13 μm poly-crystalline silicon absorbers on glass. A detailed current and resistance loss analysis is presented, supported by 2D-simulations. This way, possible levers to further improve efficiencies are identified.

## Sample Preparation

All the steps described in this section were performed at the Helmholtz-Zentum Berlin.

### Absorber Fabrication

In this section the poly-crystalline silicon thin-film absorber fabrication is described. Firstly, commercially available 1.1 mm thick alumino-silicate glasses (Corning Eagle XG) were cleaned in an alkaline process and subsequently coated with a multi-purpose layer stack of 200 nm amorphous hydrogenated silicon oxide (*SiO*_*x*_), 70 nm amorphous hydrogenated silicon nitride (*SiN*_*x*_) and 20 nm *SiO*_*x*_ without vacuum break using (PECVD). This triple (*SiO*_*x*_/*SiN*_*x*_/*SiO*_*x*_) stack will be referred to as (IL) stack and is described in more detail in refs 11–13. It serves not only as a diffusion barrier to prevent impurities from the glass to enter the silicon, but also at the same time as an anti-reflection layer, and as passivation layer. Moreover, it has to be thermally stable enough for the subsequent crystallization process and ensure proper wetting during the crystallization. On top of the IL a 15 μm thick precursor Si absorber is deposited using high deposition rate (600 nm/min) electron-beam evaporation. A thin (≈80 nm) phosphorous doped amorphous hydrogenated silicon (a-Si:H) layer is subsequently deposited by PECVD and serves as doping source. The final absorber doping density can be easily tuned varying the phosphine flow during the deposition of said thin layer.

Finally, a sacrificial *SiO*_*x*_ is deposited to ensure additional wetting and protect the sample from contamination during the conversion of the precursor from its initial amorphous-nano-crystalline state to poly-crystalline Si^14^. The core of the technology, the conversion to poly-crystalline is dubbed (LPC) and is carried out in vacuum using a line-shaped 808 nm wavelength CW laser (beam length: 52 mm, beam width: 0.3 mm) with a scanning speed of 3 mm/s. During the scan the silicon absorber is locally entirely molten and re-crystallizes along the scanning direction into elongated grains with a height as thick as the complete absorber layer, width up to millimeters and length up to centimeters^15^. The doping atoms of the thin doped a-Si:H layer are homogeneously distributed into the whole absorber during the LPC process. After LPC, stress from the glass is relieved in a rapid thermal annealing process in an oven at 950 °C.

Then, the sacrificial oxide capping layer is dissolved in 5% hydrofluoric acid and the absorber is passivated using a hydrogen plasma treatment at 600 °C for 15 min^6, 16^. Finally, it is textured using a common KOH based process with a texturing agent by GP Solar (Alkatex) that enables an isopropylic-alcohol-free process at 80 °C for around 3 min. Due to the poly-crystalline nature of the absorber, grain surfaces with {100} surface orientation of the absorber show ≈2 μm pyramids whereas on the other grain orientations the pyramids are tilted by various angles up to being almost flat^17^. After all chemical treatment described above the absorber thickness is reduced to roughly 13 μm.

### Contact System Fabrication

**Figure 1:**
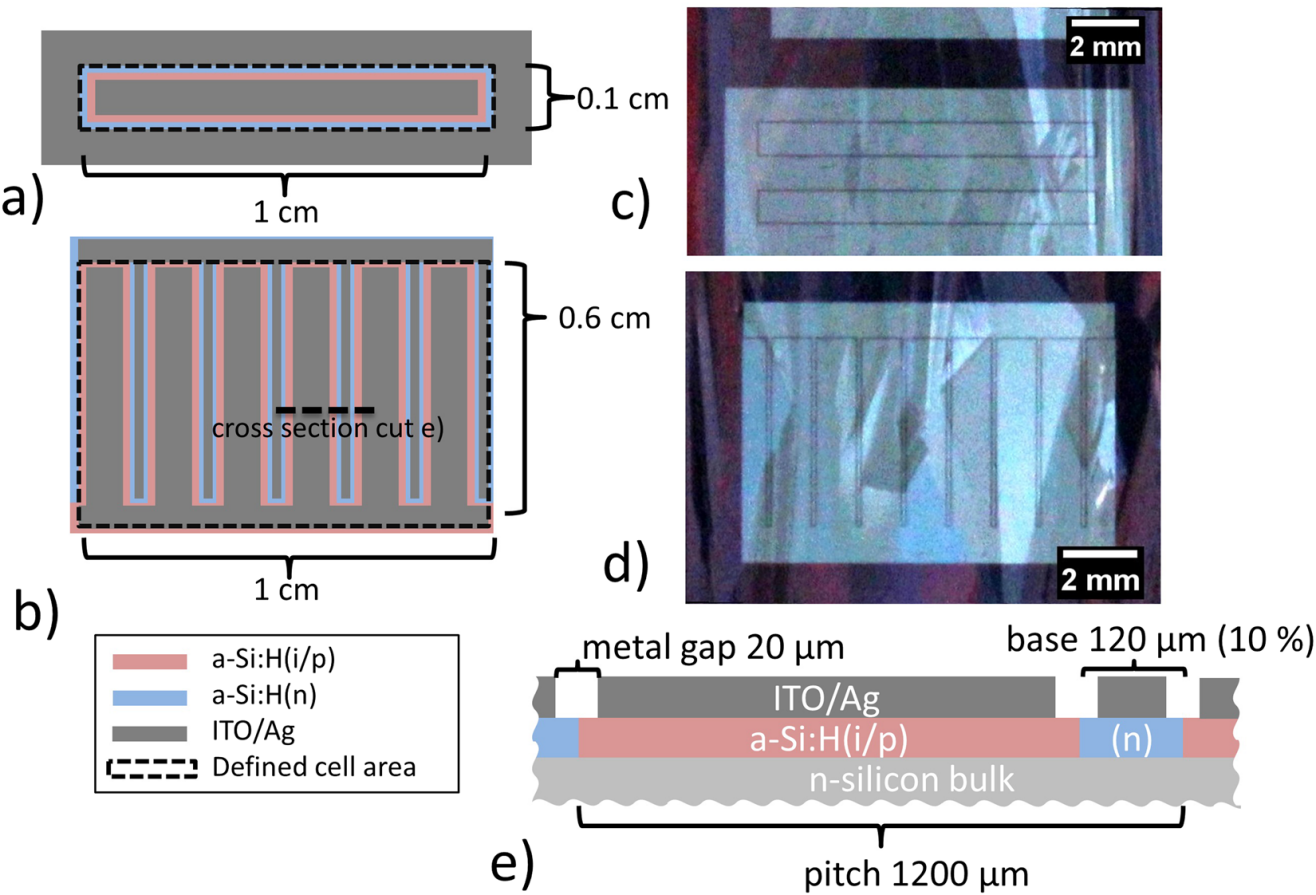
Top view from the back side of (**a**) full-emitter-cell (FEC) structure and (**b**) IBC cell. Photograph of two FEC structures (**c**) and one IBC cell (**d**) on glass from the LPC-Si layer/contact system side. (**e**) cross section of IBC cell as marked by dashed line in (**b**).

On the previously described absorbers two different cell types are fabricated. Their fabrication shall be described in the following. One cell type is an interdigitated back-contact (IBC) system featuring an amorphous hydrogenated silicon (a-Si:H)/crystalline silicon heterojunction (SHJ)^18^ with a cell size of 0.6 cm^2^ similar to the one described in ref. 19 while the other is a small rectangular test structure. This cell is also back-junction-back-contacted, but only the emitter is counted as cell area. The absorber contact lies outside of the rectangle. Hence, we call it full-emitter cell (FEC) structure. These structures are simple in their design and since the cell area is emitter-only, they provide insight into the material quality. They are much less prone to ohmic losses and thus show higher efficiencies. Since they are co-processed on the same glass-substrate with the IBC cells detailed analysis of materials and device related aspects are possible. Both cell designs are schematically depicted in Fig. 1(a,b,e) together with photographs of the respective designs in (c) (two FEC structures) and (d) (IBC). To fabricate both contact systems a standard RCA^20^ cleaning is performed on the hydrogen passivated and textured LPC absorbers. Subsequently, intrinsic (i) and boron (p) doped a-Si:H is deposited via PECVD. The layers are structured using photolithography (Resist: Microchemicals AZ4533, maskaligner: MA6 Suess microtec). Contrary to the 0.6% NaOH solution used in ref. 19, a 2.5% solution of (TMAH) was deployed for development. It is a metal-ion-free developer solution which is described by Tabata *el al*.^21^. Other developer solutions contain mobile ions (potassium or sodium), which are known to cause contamination problems in Si material^22^. After developing, the photosensitive resin defines the emitter area. Exposed areas are etched using a mixture of nitric, phosphorous, and hydrofluoric acid.

The absorber or back surface field (BSF) contact is formed using a thin layer of a-Si:H(n) deposited by PECVD. The layer is structured in the same manner as the emitter using a selective etch process similar to the one described in refs 19, 23 but using 2.5% TMAH instead of KOH or NaOH. For TMAH also heavily boron doped layers work as an etch-stop^21^. We found that TMAH etches more homogeneously on small areas. 110–120 nm of ITO sputtered at room temperature and 1.5 μm of sputtered Ag form the electrodes of both the IBC and the FEC types. They are also defined by photolithography and selective etching using a diluted mixture of NH_4_OH and H_2_O_2_ for the Ag and HCl for the ITO. A post-annealing step is performed in an oven at 200 °C for 15 min.

### Characterization

Current-voltage (*J*–*V*) curves were obtained using the solar simulator Wacom WXS-156S-L2 with AAA characteristics. The external quantum efficiency (EQE) were measured illuminating the full area using a filter-wheel setup and internal quantum efficiency (IQE) were calculated using reflection spectra measured by a PerkinElmer LAMBDA 1050 spectrometer. In addition, light-beam induced current (LBIC) and electro-luminescence (EL) mappings were recorded using self-built setups at LPVO at the University of Ljubljana^24^. The LBIC setup uses a wavelength of 638 nm (≈3 μm penetration depth in Si) and has a minimal beam width below 10 μm. 2-D simulations of the cells were realized with the photovoltaics software ASPIN3 which was developed at LPVO at the University of Ljubljana^25^.

## Results

**Figure 2:**
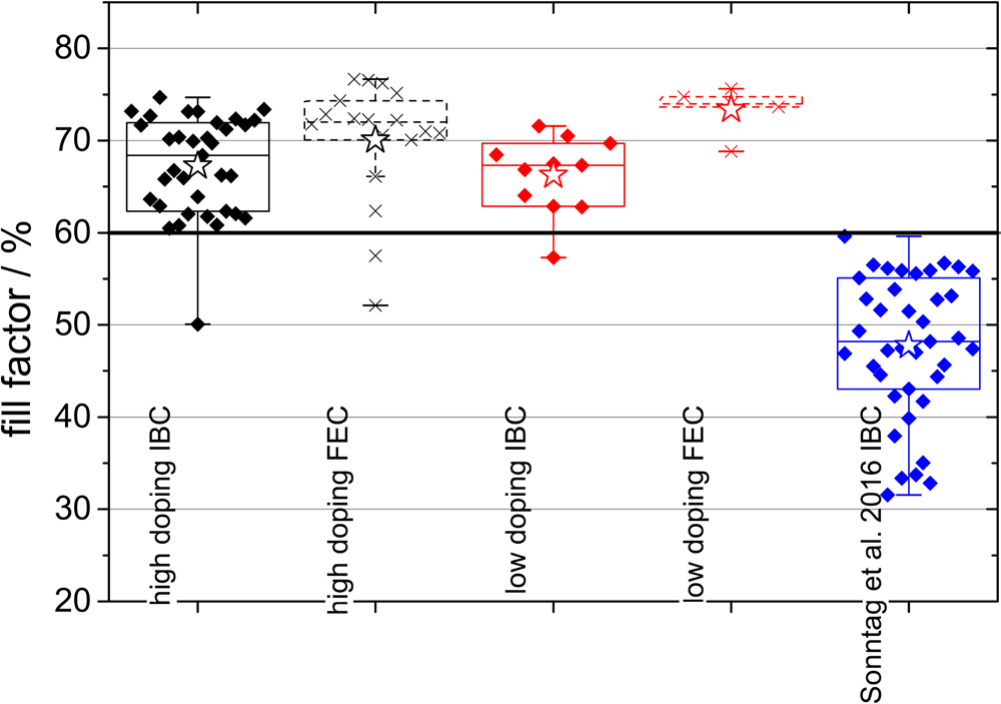
FF box plot of IBC and FEC structures of this work for high (black) and low (red) doping and FF of the cells fabricated in the course of the work from Sonntag *et al*. 2016^19^ in blue.

IBC and FEC cells were fabricated with one “high” and one “low” absorber doping density by varying the phosphine flow during the deposition of the a-Si:H doping layer. Over 30 IBC cells and around 20 FEC structures with the high doping and over 10 IBC cells and 6 FEC with the low doping were produced and characterized by the solar simulator. In Fig. 2 the FF of these cells are plotted as statistical box plots. The FF of highly doped samples are depicted in black. For IBC and FEC full diamonds and crosses, respectively, were used to represent single cells’ data. The FF for the lowly doped samples are added analogously in red. Blue diamonds represent data from ref. 19 using a slightly different IBC process that is described therein in detail. Empty stars indicate mean values. The highest values for the FF were 75% on IBC and 77% on FEC structures for the cells in this work and 59% for the cells from ref. 19.

### Mobility and Doping

Mobility and doping concentrations were determined on 12 small 5 × 5 mm^2^ samples. They were cut out of an absorber that was co-processed with some of the highly doped samples. We chose such a large number of samples to obtain sufficient statistics as grain boundary and dislocation density variations over the whole 5 × 5 cm^2^ substrates may lead to local differences in mobility and doping concentration. Also, the device geometry feature sizes are of the same order of magnitude as the typical grain sizes. Hall results yielded a mobility of (530 ± 80)cm^2^/*Vs* which corresponds to 82% of what can be calculated with the Masetti model^26^ for mono-crystalline silicon. The doping density was determined via both Hall and four point probe sheet resistance measurements. Both resulted in an *N*_*D*_ = (1.2 ± 0.2) · 10^17^/cm^3^ and *N*_*D*_ = (1.3 ± 0.4) · 10^17^/cm^3^, respectively. For the sheet resistance measurements a mobility of 80% of the mono-crystalline silicon values of the target doping density was assumed with an error of ±20% of said assumed mobility. As these values agree very well, the doping density for the lower doped samples was determined to *N*_*D*_ = (2.0 ± 0.4) · 10^16^/cm^3^ via four point probe measurement assuming the same reduced mobility (80% of mono-crystalline silicon).

### Current–Voltage Characteristics and Quantum Efficiency

**Figure 3:**
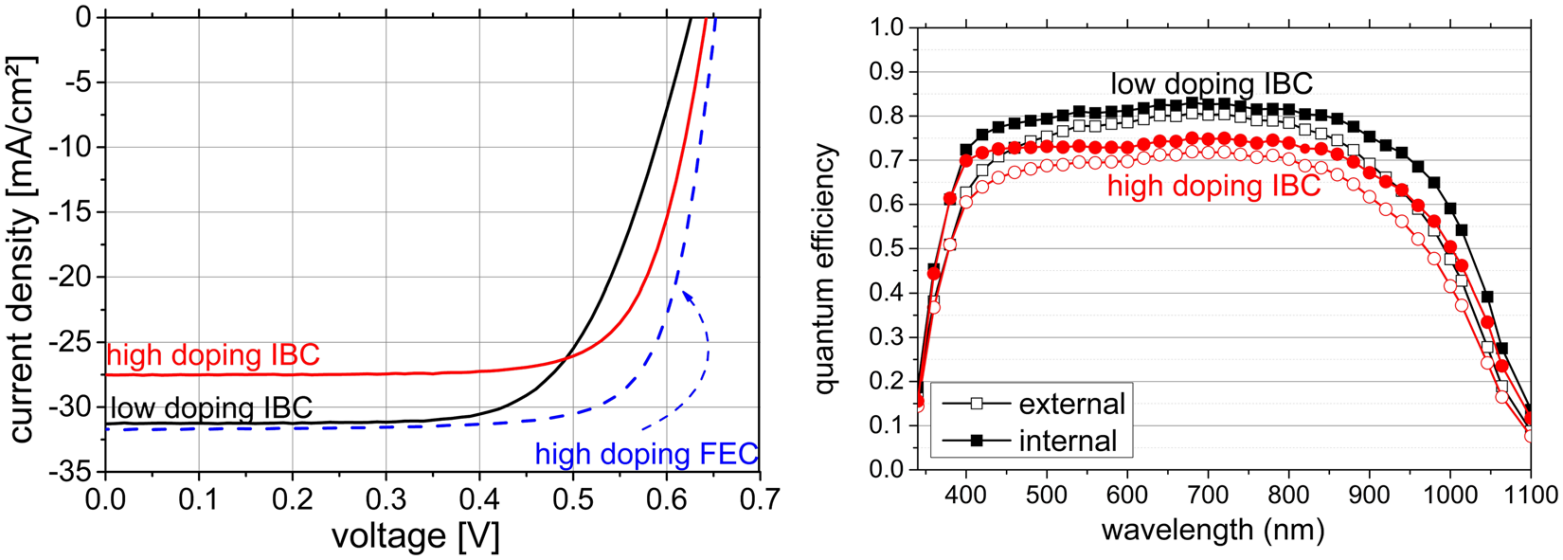
(**a**) *J*−*V* curves of the best IBC cells on LPC absorbers with high (red) and low (black) doping, respectively. The best FEC structure (blue) is added to demonstrate the potential of the absorbers. (**b**) External (open symbols) and internal (closed symbols) QE of the high (circles) and low (squares) IBC cells in 3a). FEC structure is omitted to not overload the graphic. *J*_*SC*_ determined from integrating EQE curves corresponds well within errors with solar simulator data.

In Fig. 3(a) the *J*–*V*-characteristics of the best IBC cells and the best FEC structure are plotted. They were measured with an additional light trapping anti-reflection foil (ARF) by DSM advanced surfaces^27^ placed on the glass. The exact parameters are listed in Table 1. The *V*_*OC*_ of the lowly doped IBC is 16 mV and the FF ≈ 7%_abs_ lower than that of the highly doped cell, whereas its short-circuit current density (*J*_*SC*_) is around 4 mA/cm^2^ higher. The current of the FEC is the highest with 31.7 mA/cm^2^, since only the emitter area is counted as cell area. A deeper analysis of the current densities via LBIC follows in the section about current analysis. *V*_*OC*_ of all the devices produced is well above 610 mV for the higher doped absorbers and above 590 mV for the lower doped ones (statistics not shown). A quarter of the cells was again characterized under the solar simulator after 4 months and no degradation was observed (not shown). QE of the two best IBC cells are shown in Fig. 3(b). External QE using open symbols and internal QE using closed ones and red circles and black squares for the highly and lowly doped cell, respectively. *J*_*SC*_ determined by integrating the EQE and correspond well within errors with the ones obtained by solar simulator. The maximum of the IQE are 75% and 83% for high and low doping cells, respectively.

**Table 1:** Data from *J*–*V* curve in Fig. 3(a).

cell type	*J*_*SC*_/mA/cm^2^	*V*_*OC*_/V	FF/%	η/%
low doping IBC	31.3	626	67.2	13.2
high doping IBC	27.5	642	74.7	13.2
high doping FEC	31.7	652	77.0	15.9

### Current analysis

**Figure 4:**
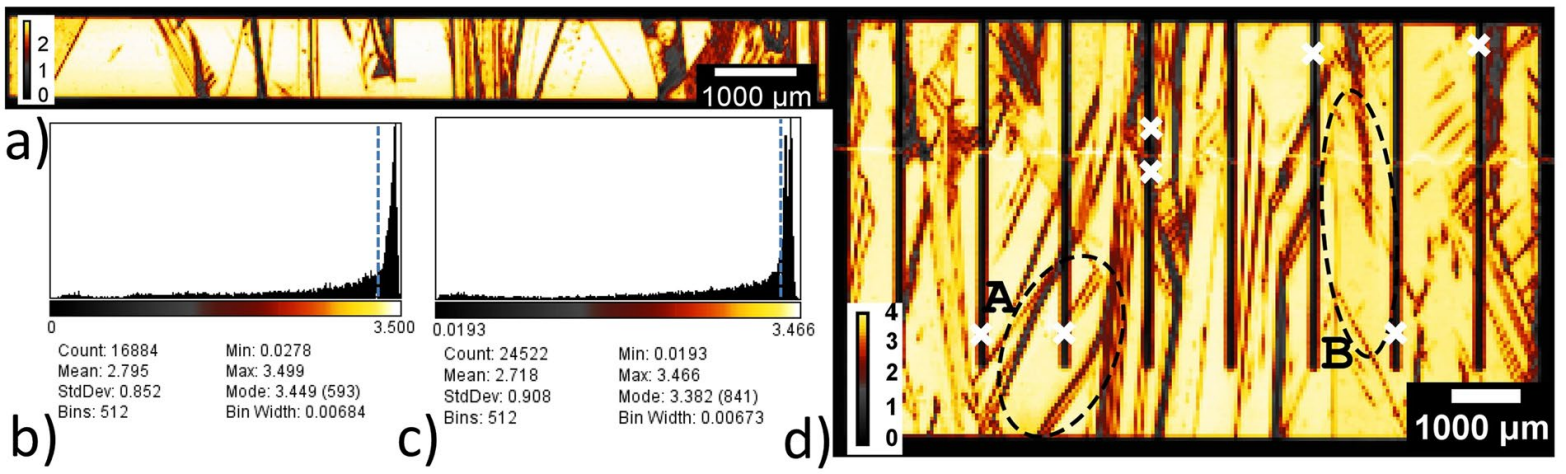
(**a**,**d**) LBIC recordings (*λ* = 638 nm) and (**b**,**c**) histogram of all measured pixel values. (**a**,**b**) FEC structure (25 μm step), (**c**,**d**) IBC cell (50 μm step). Areas of reduced collection stem from absorber contact area (regular pattern) or GB and dislocations. The bright line at about two thirds height of IBC image (**d**) is a measurement artifact from an irregularity in the glass not affecting performance. White crosses mark locations where local effective diffusion lengths were fitted from line-scans. Dashed ellipses mark areas for comparison to EL and microscope images in Fig. 6. Values above blue dashed lines in (**b**,**c**) mark values that were used for current loss evaluation.

LBIC maps were recorded from the glass side of one good quality highly doped IBC cell (efficiency 13.0%) and one good highly doped FEC structure (efficiency 15.4%). The images are shown in Fig. 4(a,d).

**Figure 5:**
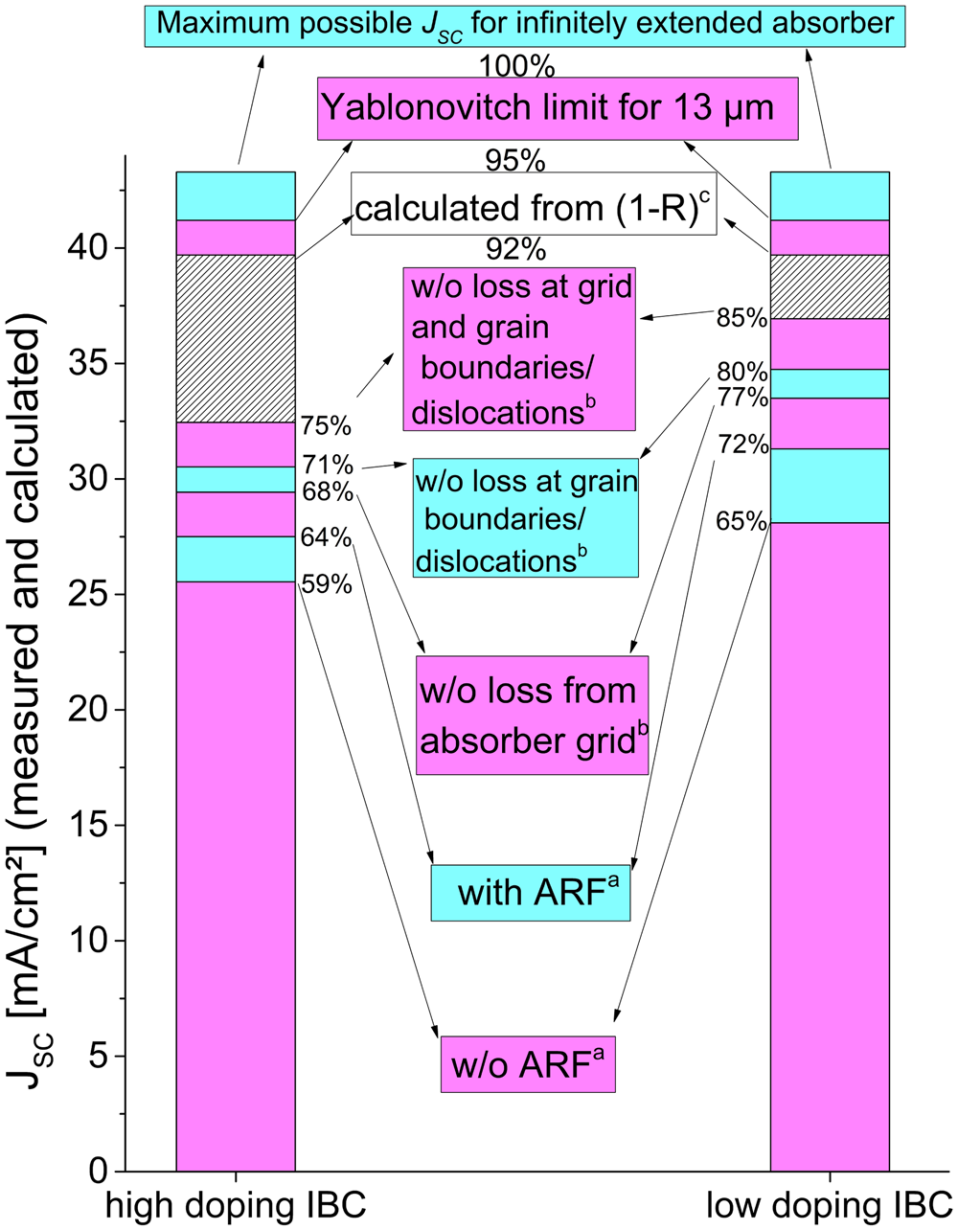
Current loss analysis for the best highly and lowly doped IBC cells. (**a**) Measured by solar simulator. (**b**) Measured *J*_*SC*_ values corrected with calculated loss fractions due to current collection inhomogeneities obtained from LBIC measurements. (**c**) Measured by UV/VIS spectrometer. Rest: calculated from literature data. Hatched areas: loss by bulk and interface recombination and parasitic absorption.

Areas of high signal (yellow-white) indicate high current collection; black areas indicate locations of low-to-zero collection. Two major loss mechanisms can be determined from the images. Firstly, at GB and dislocations which run all through the LPC absorber, the collection is reduced. Secondly, the current collection underneath the absorber contact fingers for the IBC cell is lower due to an insufficient lateral diffusion length for the minority charge carriers (holes). This phenomenon is often called electrical shading. This can be especially detrimental for n-type absorbers as the minority charge carriers exhibit lower mobilities, hence also shorter diffusion lengths, compared to the minorities in p-type (electrons). The relative loss caused by the two mechanisms (electrical shading and GB/dislocations) can be estimated from the LBIC measurements themselves. Histograms (Fig. 4(b,c)) of all the measured data points (pixels in the image) were evaluated to this end. First, a weighted average of the best 70% of the histogram values was calculated (all values above blue dashed line in Fig. 4(b,c)). This ratio was chosen, as 70% of the whole cell area seems to be a sufficient sample area and the error resulting if we would instead use the best 70 ± 10% values is negligible for this kind of estimate. Assuming this 70% area to be the average collection signal over the whole cell area (100% instead of 70%) gives a hypothetical value for a cell without GB/dislocation for the FEC cell. For the IBC cell we first excluded the values around the absorber grid (not shown). Under this assumption 11% of current is lost for the IBC cell and 14% for the FEC structure. If we now include the values around the grid of the IBC and replace them by the calculated averaged mean of the top 70% values we can make an estimate of the current reduction due to the absorber contact grid issues (electrical shading). It yielded 7%. We used these numbers (7 and 11% loss) to derive hypothetical *J*_*SC*_ values for the best highly and lowly doped IBC cells (Table 2 and Fig. 5). Furthermore, a maximum possible *J*_*SC*_ imposed by the optical design of the cell was calculated from reflection data using UV/VIS spectroscopy convoluted with the AM1.5 g reference spectrum and an IQE = 1. This hypothetical maximum Jsc yielded 39.7 mA/cm^2^ for the wavelength interval from 300–1100 nm and is 1.5 mA/cm^2^ lower than the maximum potential current of a 13 μm thick Si cell calculated assuming a path length enhancement factor of 4n^2^, the so-called Yablonovitch limit^28^. As a reference, also the maximum possible *J*_*SC*_ for an infinitely extended Si solar cell is added in Table 2 and Fig. 5.

**Table 2:** Current analysis of best highly and lowly doped IBC cells (all in mA/cm^2^).

Saturation current density mA/cm^2^	High doping IBC	Low doping IBC
maximum possible for infinitely extended absorber	43.3	43.3
Yablonovitch limit for 13 μm^28^	41.2	41.2
calculated from (1-R)^c^	39.7	39.7
without loss from absorber grid and loss at GB/dislocations^b^	32.5	36.9
without loss at GB/dislocations^b^	30.5	34.7
without loss from absorber grid^b^	29.4	33.5
with ARF^a^	27.5	31.3
without ARF^a^	25.5	28.1

^a^Measured by solar simulator. ^b^Calculated from LBIC measurements. ^c^Measured by UV/VIS spectrometer. Rest: calculated from literature data.

5% of current are lost when using an absorber of merely 13 μm. The difference between the 39.7 mA/cm^2^ from reflection data and the estimated value for a dislocation-free and grid-loss-free IBC cell represents all other losses occurring in the respective cells and are depicted in the graph by the hatched areas. Among them are parasitic absorption (in glass, IL, ITO, Ag and a-Si:H layers) and bulk and interface recombination losses. These left-over losses are 6.3 and 2.4 mA/cm^2^ for the high and low doping cells, respectively. Since the cells were identical except for the absorber doping, the difference between the left-over losses indicates a difference in bulk and/or interface recombination. The loss from parasitic absorption in glass, IL, a-Si:H layers, Ag and ITO was roughly estimated using the PVlighthouse software wafer ray tracer^29^ and n,k data from refs 30, 31. The typical stack described in the experimental part including the KOH texture was simulated and the parasitic absorption extracted and integrated over the AM1.5 g spectrum from 300–1100 nm. It resulted in 0.94 mA/cm^2^. For the sake of completeness and to underline the importance of light-trapping for thin-film Si solar cells such as the LPC-Si cells from this work, the *J*_*SC*_ that was measured under the solar simulator without the light-trapping foil anti-reflection foil (ARF) by DSM Advanced Surfaces is also added in Table 2 and Fig. 5. This represents a cell which is only one-sidedly textured. The back-side KOH texture is still included.

### Series Resistance Analysis

Series resistances for the best two IBC cells and the best FEC structure (cp. Fig. 3) were calculated comparing dark with illuminated *J*–*V* curves according to the method described in ref. 32. They are listed in Table 3. This series resistance can be subdivided into the components of base resistivity, resistivity in Ag fingers and busbars, and contact resistance of n-type and p-type contacts, respectively. All of them can be accessed using a combination of measured quantities and geometric calculations and are also listed in Table 3. More details on the calculations can be found in ref. 33 for general contact systems, and more specific for an IBC system in ref. 19. To determine the base resistivity sheet resistance measurements on various spots on the textured absorber before RCA cleaning were done. They yielded on average 50 Ω/□ and 300 Ω/□ for the highly and lowly doped sample, respectively. Accordingly, the base resistivity for lower dopant concentration is 6 times higher than for the highly doped cells. The different geometries of IBC cells and FEC cause also a difference in base resistivity. To determine the (n) and (p) contact resistances, test structures were co-processed on highly doped small wafer pieces similar to the ones described in ref. 34. They were evaluated using the transfer length method (TLM)^35^. We used wafer pieces and not LPC absorbers for the contact resistance determination to rule out any local GB/dislocation effects and/or local variations in the mobility and to ensure a certain degree of comparability with other similar contact resistances of the wafer community^36, 37^. The contact resistance yielded (60 ± 10) mΩcm^2^ for a stack of c-Si(n)/a-Si:H(n)/ITO/Ag representing the (n) contact resistance, and (335 ± 70) mΩcm^2^ for the stack c-Si(p)/a-Si:H(i/p)/ITO/Ag representing the (p) contact resistance. Considering a contact area fraction for the minority (p) contact and majority (n) contact of 88% and 8.6% we get a contribution to the series resistance of about 380 and 690 mΩcm^2^, respectively.

**Table 3:** Series resistance contributions to various cells and the series resistance determined by comparing dark and illuminated *J*–*V* curves.

Series resistance in Ωcm^2^	High doping IBC	Low doping IBC	High doping FEC
(n)-contact resistance (whole cell area)	0.69	0.69	—
(p)-contact resistance (whole cell area)	0.38	0.38	0.34
Base	0.05	0.30	0.004
Ag fingers and busbars	0.034	0.034	0.025
Sum of calculated contributions	1.16	1.41	0.36
Whole cell *R*_*S*_ determined by comparing dark vs illuminated *J*–*V* curve	1.32 ± 0.21	2.97 ± 0.19	0.68 ± 0.19

**Figure 6:**
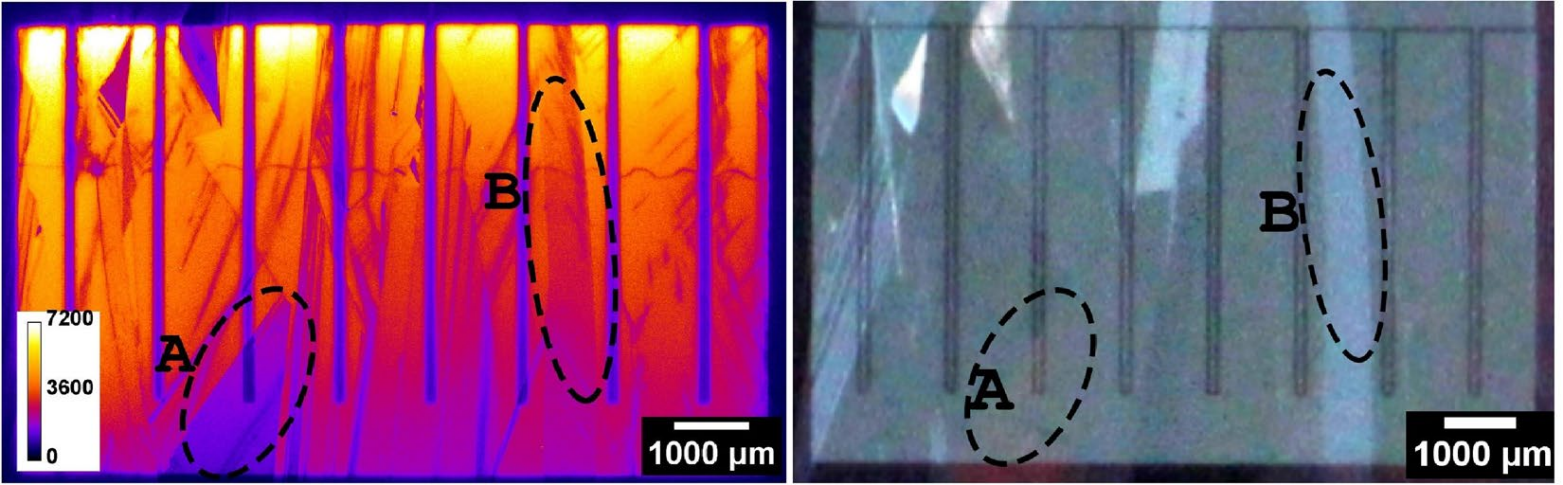
(**a**) EL image of the same cell as depicted in Fig. 4. Image was recorded at V = 0.67 V, I = 0.015 A. Counts in arbitrary intensity units. Same measurement artefact from a glass irregularity as in Fig. 4 is seen as wobbly line perpendicular to the grid fingers. Local intensity variations stem from contact resistance or lifetime variations. Optical microscope image of the back side of the same cell. Image was mirrored to directly compare electro-optical effects (**a**) with pure optical effects (**b**). Dashed ellipses mark areas used for evaluating optical, series resistance and lifetime effects together with areas in Fig. 4(d).

All series resistance contributions are depending on the contact system geometries. The geometries we chose were a pitch of 1.2 mm, an emitter(p)-to-pitch ratio of 88%, and a cell size of 0.6 × 1 cm^2^. There is always a trade-off between all the contributions of an IBC system assuming a constant contact resistance (in Ωcm^2^). If the (p)-contact area fraction is increased, the contribution to the series resistance from the (n)-contact area rises. While keeping a constant emitter-to-pitch ratio, increasing the pitch will reduce the contact resistance fraction but the contribution from the base will rise. The series resistance for the whole device determined by comparing dark and illuminated *J*–*V* curves was higher than the sum of the calculated constituents in all cases. This is due to fact that the contact resistance was determined on planar wafer pieces, while the actual contact resistance on the LPC-Si material changes with underlying surface orientation and the base resistivity has local changes induced by variations in dislocation density within the absorber. This can be seen by looking at the EL and optical microscope image depicted in Fig. 6. The same cell as for the LBIC image in Fig. 4 was used to record the image. Areas of high EL emission are yellow-to-white, areas of low emission blue-to-black. Note that grains with reduced EL emission may be darker due local series resistance effects, but as well due to a reduced lifetime but not different surface morphology. The latter means different crystal orientations cause the anisotropic KOH texturing to modify the surface differently. The LPC-Si back-side contains areas of 2 μm upright pyramids ({100} initial crystal orientation) up to areas that are almost flat (near or close to {111} initial). The former areas scatter light in all directions and are consequently darker in the optical microscope image, which is taken from the back-side), while the latter ones preferentially reflect light one-directional and are brighter in the optical microscope image (e.g. area B). Since the luminescence signal is already emitted statistically in random directions at a given point in the bulk, an additional scattering or specular reflection at the ITO/Ag back-side has no influence on the EL signal. What we see are only electrical effects. The latter include series resistance and lifetime (diffusion length) variations within the absorber. The LBIC image in Fig. 4d) helps distinguishing between them. Exemplarily, two areas A and B are marked in the LBIC image as well as in the EL and optical microscope image in Fig. 6. Both areas are affected by a high series resistance. This can be stated, since the LBIC image is not affected by series resistance effects due to the low currents flowing (~*μ*A) and not affected by optics, i.e. the back-side texture (penetration depth of the LBIC beam ~3 μm). Both areas A and B show a high current collection in LBIC. This means their diffusion length does not differ much from the surrounding areas of high collection, so no diffusion length problem is evident. Since they are not darker as surrounding areas on the optical light microscope image (no preferential out-scattering), but clearly darker in EL, these areas are indeed affected by higher local series resistance.

### Diffusion Length Evaluation

Local effective minority charge carrier diffusion lengths were determined according to the method described in ref. 38 from the LBIC recording depicted in Fig. 4. The method is based on the fact that minorities (holes) that are generated by the LBIC beam under the absorber contact have to travel laterally to the emitter and partly recombine on the way. A measure for this recombination fraction is the effective diffusion length which takes bulk and surface effects into account. Line-scans across the absorber contact at places indicated by a white cross were measured with a resolution below 10 μm and a step width of 5 μm (not shown). The collection signal *p*(*x*) at the absorber contact was fitted according to

1 $$p(x)=({e}^{\frac{-(x-{x}_{1})}{{L}_{diff}}}+{e}^{\frac{(x-{x}_{2})}{{L}_{diff}}})\mathrm{.}$$

Parameters *x*_1_ and *x*_2_ describe the shift in LBIC scan direction of the exponential functions relative to each other, effective minority charge carrier diffusion length (*L*_*diff*_) is the effective minority carrier diffusion length. All the fits had very good quality and ranged from (14–19) μm with the exception of one that was below 7 μm. Worth noting is that this diffusion length takes the bulk as well as both front and back side interfaces into account. Hence, we call it an effective diffusion length. A separation into bulk and interface effects is not possible at this point.

### Simulation

**Figure 7:**
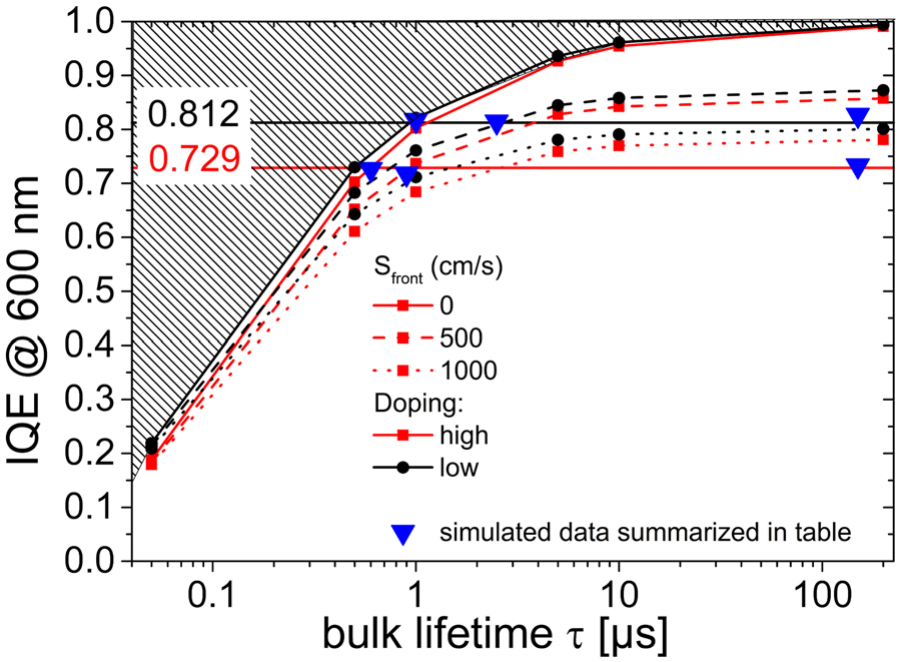
Simulated IQE at 600 nm of high (red squares) and low (black circles) doping IBC structures that were implemented into ASPIN3 as a function of bulk lifetime. IQE were simulated for different front surface recombination velocities (solid lines: 0 cm/s, dashed: 500 cm/s, dotted: 1000 cm/s). Measured IQE values at 600 nm of the two IBC cells from Fig. 3 are implemented as horizontal lines. Hatched area above the 0 cm/s lines is outside the parameter space for valid solutions for any set of lifetime and recombination velocity to reach an IQE value in this area. Face-down triangles represent simulated cells with IQE matching the ones measured for the three cases: bulk limited, intermediate and interface limited in Table 4.

Accessing typical absorber parameters such as the lifetime via QSSPC measurements is not possible with LPC-Si absorbers because of the low sample volume resulting in too low signal-to-noise ratios. Simulations, however, offer a good alternative to estimate lifetime values and further to distinguish between the influence of bulk and interface. To this end, a structure based on the geometries of the investigated cells with high and low doping was implemented into ASPIN3 a dedicated numerical solver for the semiconductor equations. Mobilities of 80% of c-Si according to the Masetti modell^26^ were used. Since the optical model included in ASPIN3 is limited to planar layers and the IBC cells in this work were textured, we chose to evaluate the IQE of measured and simulated data for better comparison. In Fig. 7 we plotted the IQE values at 600 nm for three different front surface recombination velocities (*S*_*front*_) (i.e. interface qualities) as a function of the bulk Shockley-Read-Hall lifetime (τ_*bulk*_) (note the logarithmic scale). The back surface recombination velocity is assumed to be zero (or negligible) due to the passivated a-Si:H contacts. Dots are simulated values; lines are guides to the eye. High doping values are depicted as red squares, low doping values as black circles. The maximum achievable IQE for a given bulk lifetime is set by the *S*_*front*_ = 0 cm/s line. The hatched area above is out of the possible parameter space. It can clearly be seen that with higher *S*_*front*_, the maximum achievable IQE (i.e. for long lifetimes over 100 μs) sinks. To compare simulation results with the experiment, the measured IQE values for highly (72.9%) and lowly (81.2%) doped IBC were added as horizontal lines. All points on the horizontal lines after they cross their respective *S*_*front*_ = 0 lines represent a correct parameter set to simulate the respective cell. If *S*_*front*_, however, exceeds a certain value it will never reach the respective measured IQE value. For example no τ_*bulk*_ exists for an *S*_*front*_ = 1000 cm/s for the low doping to reach 81.2% IQE at 600 nm. This way we can give an easy estimate for the maximum possible *S*_*front*_ for each doping. It is around 800 cm/s for the low, and 1400 cm/s for the high doping, respectively. We call this the interface limited case. Analogously the lifetimes for each doping must be higher than 0.6 μs and 1.0 μs for high and low doping IBC, respectively, because lower lifetimes would be outside of the parameter space (*S*_*front*_ = 0 already means perfectly passivated front side). We call this the bulk limited case. Additionally, one intermediate case was examined. All three cases are listed in Table 4 and also added in Fig. 7 as blue face-down triangles. *J*_*SC*_ was omitted in the table, since it is not expedient to compare textured experimental and planar simulated cells. Simulated *R*_*P*_ was above 3 kΩcm^2^, so that any influences on the *V*_*OC*_ can be ruled out. Of course a reduced *J*_*SC*_ on the planar simulated cells reduces also *V*_*OC*_ to some extent. However, other loss mechanisms e.g. at the a-Si/LPC-Si interface were not taken account of in the simulation, so the *V*_*OC*_ values should be regarded with caution. Keeping this in mind, the comparison with actual measured *V*_*OC*_ in case of the high doping cell of 642 mV is quite satisfying. The simulated diffusion lengths are higher than the measured values of (14–19) μm. This may be due to insufficient measurement statistics. The method to extract the diffusion length can only be applied at the absorber contact because a signal drop is needed for the evaluation. Diffusion lengths might be different underneath the emitter.

**Table 4:** Overview over simulated cells with same IQE at 600 nm as best IBC cells on high and low doping absorbers.

Simulated case	High doping (IQE(600 nm) = 72.9%)				Low doping (IQE (600 nm) = 81.2%)			
	*S*_*front*_ (cm/s)	τ_*bulk*_ (μs)	*L*_*diff*_ (μm)	*V*_*OC*_ (mV)	*S*_*front*_ (cm/s)	τ_*bulk*_ (μs)	*L*_*diff*_ (μm)	*V*_*OC*_ (mV)
Bulk limited	0	0.6	18.2	652	0	1.0	25.2	617
Medium	500	1.4	22.4	655	500	2.5	28.4	623
Interface limited	1400	150	22.6	662	800	150	31.9	630

Three cases were simulated: bulk limited, interface limited, and one intermediate case. *S*_*front*_, τ_*bulk*_ input parameters are listed with their respective resulting *L*_*diff*_.

## Discussion

The electron mobility with values as high as 82% of mono c-Si mobility confirm the high LPC-Si absorber quality in accordance with previously published data^6, 7, 39, 40^. 13.2% conversion efficiencies were demonstrated on two cells on distinctly different doping densities being almost one order of magnitude apart. The cells show different limitations. The lowly doped cell reveals a high short circuit current density of 31.3 mA/cm^2^ while the highly doped cell only shows 27.5 mA/cm^2^. This lower current is induced by a lower diffusion length as seen from simulated IQE for all three cases (bulk, intermediate and interface limited). Since the lower diffusion length is an inherent material property, the ARF placed on the glass for external additional light trapping does not affect this difference in *J*_*SC*_. Assuming the same fraction of electrical shading of 7% due to the absorber contact grid, the difference in *J*_*SC*_ loss due to surface and bulk recombination is 4.4 mA/cm^2^ (cp. Fig. 5). This loss difference was determined by a detailed analysis and corresponds directly to the actually observed *J*_*SC*_ difference from *J*–*V*-characteristics of the solar simulator. The *J*_*SC*_ is on a high level for both cells considering such thin absorbers due to an efficient light in-coupling and light trapping by a front side anti-reflection foil, a *SiN*_*x*_ index matching layer and a KOH textured pyramidal back-side and an ITO/Ag rear reflector. From the evaluation of LBIC measurements diffusion lengths of (14–19) μm were determined for the highly doped absorber compared to (18.2–22.6) μm for three simulation cases. The difference may stem from the fact that via measurements diffusion lengths can only be accessed underneath the absorber contact and not underneath the emitter. The lower doping yielded simulated diffusion lengths of (25.2–31.9) μm indicating better material quality. The current losses due to effects at GB and dislocation rich areas were estimated to be around 11–14% (from values of both IBC cell and FEC structure in Fig. 4). With advanced crystallization techniques such as described in ref. 39 or more effective hydrogen plasma passivation of the absorber^13^ this value could be reduced in the future, but is at the moment a major loss factor. The loss translates into 4.4 mA/cm^2^ loss for the FEC structure and 3.0 and 3.4 mA/cm^2^ for highly and lowly doped IBC, respectively. On top of that, the IBC suffer from electrical shading losses of about 7% determined by evaluating the LBIC histogram. The losses are a function of the pitch, absorber contact finger width and the effective diffusion length which takes bulk and surface effects into account. The absorber contact at the back-side has no intrinsic a-Si:H layer beneath the a-Si:H(n). By adding such a layer, the back-side surface passivation could be elevated to potentially reduce electrical shading. The last lever to reduce the electrical shading losses is the contact system, namely pitch and absorber contact finger width. At the moment the emitter-to-pitch ratio is around 90%. It could be increased to decrease the electrical shading losses. However, at the expense of fill factor. Especially the contact resistance contributes largely to the overall series resistance. Around 50–60% of the calculated total series resistance (depending on the doping) is due to the absorber contact. Especially, the low doping IBC cell suffers from a low FF of 67.2% compared to the 74.7% of the high doping one. This is mainly due to the higher series resistance of (2.97 ± 0.19) Ωcm^2^ compared to (1.32 ± 0.21) Ωcm^2^ of the highly doped IBC cell. The difference in summed up constituents of the series resistance and the actually measured one by comparing dark and illuminated *J*–*V*-characteristics is strikingly more for the low doping cell than for the high doping cell. This might due to a higher contact resistance of the lowly doped cell. The contact resistance entering the calculations was evaluated on co-processed wafer pieces and not on the respective LPC absorbers themselves. It can also be explained partly by local series resistance differences caused by different grains and their different orientations. From EL images (cp. Fig. 6) we can see that some grains are almost electrically dead, resulting in variations of the series resistance from cell to cell depending on the grains they are composed of. A more detailed investigation could reveal, which type of grains have higher contact resistance or are electrically dead to help avoid creating them during the LPC process.

The highly doped cell has a 16 mV higher *V*_*OC*_ than the lowly doped one (Table 1). This is possible despite its shorter diffusion lengths solely due to the higher absorber doping as we will show by the following simple picture. From literature it is known that the *V*_*OC*_ follows

2 $${V}_{OC}\approx \frac{kT}{e}\,\mathrm{ln}(\frac{{J}_{SC}}{{J}_{0}}),$$

with the Boltzman constant *k*, the absolute temperature *T*, the elementary charge *e*, the short circuit current density *J*_*SC*_, and the saturation current density *J*_0_. The latter is a function of the doping density and the minority charge carrier diffusion length *L*_*diff*_ and can be expressed as

3 $${J}_{0}=e\cdot {n}_{i}^{2}(\frac{D}{{L}_{diff}\cdot {N}_{D}}),$$

with the constant of diffusivity for minorities (holes) *D*. For the highly doped cell *N*_*D*_ is about an order of magnitude higher. The diffusivity for minorities only depends on their mobility, which is on a generally high level and does changes by less than 20% within the one order of magnitude of *N*_*D*_^26^. An upper estimate for the change in *L*_*diff*_ is about 40% (Table 4). Using these estimates in Equations (2) and (3), we see directly that the breakdown in absorber and interface passivation leading to a lower *L*_*diff*_ for the higher doping cell is over-compensated by the 1000% higher doping concentration itself and thus leads to an increase in *V*_*OC*_ (even if we include also the 13% difference in *J*_*SC*_ into our estimation).

The FEC structure has an overall better performance than the IBC cells due to various reasons. The electrical shading losses are canceled out since they lie outside of the defined cell area. The smaller cell design causes a reduced series resistance. Furthermore, the cell is quite simple so it is less prone to process issues. Lastly, it is 6 times shorter than the IBC counterpart making it more likely to cover only areas of grains with a favorable contact resistance and scattering properties. All this leads to the efficiency as high as 15.9% to effectively demonstrate the future potential of the LPC-technology.

## Conclusions

In this work, we presented our newest advances in cell efficiency on 13 μm thin liquid-phase crystallized absorbers of on glass. Due to the improved etching process involving the metal-free TMAH, an integrated PECVD interlayer stack between glass and LPC-Si and optimized contact system geometries a power conversion efficiency of 13.2% on two differently doped absorbers could be demonstrated. The low doping IBC cell had an *N*_*D*_ = (2.0 ± 0.4) · 10^16^/cm^3^ and the high doping IBC cell *N*_*D*_ = (1.3 ± 0.4) · 10^17^/cm^3^. Both cells had an area of 0.6 cm^2^ and the contact system was identical. While the low doping cell was limited by a FF of 67.2% due to a higher series resistance (compared to 74.7% for high doping), the high doping cell was limited by a lower *J*_*SC*_ of 27.5 mA/cm^2^ (31.3 mA/cm^2^ for low doping). Both cells were limited by recombination at GB and dislocations. From 2D-simulations we conclude that more development should be focused on improving the interlayer/LPC-Si interfaces, as they limit the IQE potential drastically. Simulated diffusion lengths show that thicker absorbers can be used in the future for low doping densities to harvest more light from the AM1.5 g spectrum. Furthermore, better control of the crystallization process will be necessary to mitigate losses at GB and dislocations that are right now a major source of current loss, as the evaluation of light beam induced current measurements revealed. Lastly, small 0.1 × 1 cm^2^ full-emitter cell structures were designed to demonstrate the potential of the LPC technology by circumventing some of the limitations of the IBC cells. The highest efficiency of those structures was 15.9%. This efficiency shows how LPC-cell efficiencies can evolve in the future by reducing sources of losses like electrical shading, GB and by reducing the series resistance further.
